# PReS-FINAL-2218: Treatment of colchicines resistant FMF: experience of a pediatric center in Turkey

**DOI:** 10.1186/1546-0096-11-S2-P208

**Published:** 2013-12-05

**Authors:** F Kara Eroglu, Y Bilginer, F Ozaltin, R Topaloglu, S Ozen

**Affiliations:** 1 Pediatric Nephrology-Rheumatology Department, Hacettepe University Faculty of Medicine, Ankara, Turkey

## Introduction

Familial Mediterrenean fever (FMF) is an autoinflammmatory disorder which result from mutations in MEFV gene and characterized by dysregulated inflammasome activation and IL1β secretion. At least 5% of FMF patients do not respond to colchicine.

## Objectives

To evaluate the characteristics of colchicine resistant children and adolescents and present our initial treatment results.

## Methods

FMF resistance was defined as having ≥2 attacks in a month and persistently high CRP and SAA levels during the attack free period, in spite of adequate colchine dose. All patients were homozygous or compound heterozygotes for MEFV mutations. One patient who is on anakinra treatment has HIDS mutation as well. All continued colchicine treatment at a mean dose of 0,04 ± 0,01 mg/kg.

## Results

Eleven patients with mean age of 12,7 ± 7,7 years (median 14, ranging 1,5-23 years) were studied. These patients were on colchicine treatment for a mean of 5,5 ± 4,2 years. A total of 7 patients were started anakinra, however since 2 had local reactions and 2 was unresponsive; they were switched to canakinumab. At this time a total of 8 patients are now being treated with canakinumab with a mean duration of 10,8 ± 6,8 months and 3 patients with anakinra with a mean duration of 19,6 months and they all have normal acute phase reactants.

In one patient initially etanercept was used, she initially responded well but after 6 months, she became resistant and switched to anakinra. She was also well with anakinra but after 8 months, she also became resistant to anakinra despite increasing dose up to 6 mg/kg and started canakinumab. Now she has been well with canakinumab for 6 months.

Except two patients who had local reactions with anakinra, there were no side effects.

## Conclusion

Anti IL1 treatment is beneficial in FMF patients who are resistant to colchicine and can be used safely. These patients may also become resistant to anti TNF or anakinra treatment.

## Disclosure of interest

None declared.

